# Down-Regulation of microRNA-132 is Associated with Poor Prognosis of Colorectal Cancer

**DOI:** 10.1245/s10434-016-5133-3

**Published:** 2016-02-11

**Authors:** Yukako Mokutani, Mamoru Uemura, Koji Munakata, Daisuke Okuzaki, Naotsugu Haraguchi, Hidekazu Takahashi, Junichi Nishimura, Taishi Hata, Kohei Murata, Ichiro Takemasa, Tsunekazu Mizushima, Yuichiro Doki, Masaki Mori, Hirofumi Yamamoto

**Affiliations:** 1 Department of Surgery, Gastroenterological Surgery, Graduate School of Medicine, Osaka University, Suita City, Osaka Japan; 2 DNA-chip Development Center for Infectious Diseases, Research Institute for Microbial Diseases, Osaka University, Suita City, Osaka Japan; 3 Department of Surgery, Suita Municipal Hospital, Suita City, Osaka Japan; 4 Department of Molecular Pathology, Division of Health Sciences, Graduate School of Medicine, Osaka University, Suita City, Osaka Japan

## Abstract

**Background:**

Given the role of microRNA in colorectal cancer (CRC) progression, we explored the association between microRNA (miRNA) expression and CRC-related prognosis.

**Methods:**

Three types of tissue samples (primary CRC lesions without liver metastasis, primary lesions with liver metastasis, and liver metastatic tissues) were used for miRNA profiling to identify differentially expressed miRNA. Quantitative real-time PCR was used to examine miRNA expression in CRC cells and in tumor tissues.

**Results:**

MiR-132 was significantly down-regulated in primary CRC tissues with liver metastasis and liver metastatic lesions compared to primary lesions without liver metastasis. Multivariate analysis for overall survival indicated that low miR-132 expression was an independent prognostic factor for CRC patients (overall survival *P* = 0.040, disease-free survival *P* = 0.015). Ectopic expression of miR-132 significantly inhibited cell proliferation and cell invasion. The luciferase reporter assay revealed that anoctamin 1 (ANO1) was a direct target of miR-132. Kaplan–Meier survival curves showed that high ANO1 expression was a significant prognostic factor for overall survival of patients with CRC (*P* = 0.0344).

**Conclusions:**

Down-regulation of miR-132 is associated with poor prognosis in CRC. ANO1 could be one of the crucial targets of miR-132 in CRC.

**Electronic supplementary material:**

The online version of this article (doi:10.1245/s10434-016-5133-3) contains supplementary material, which is available to authorized users.

Colorectal cancer (CRC) is one of the most prevalent carcinomas throughout the world.^1^ The liver is the most common organ for distant CRC metastasis, and liver metastasis is the leading cause of cancer-related death in CRC patients.^2^ Approximately 30–50 % of CRC patients develop local tumor recurrence or distant metastasis after curative resection of the primary lesion.^3^,^4^ New diagnostic markers that can detect early metastasis or predict the risk of metastasis in CRC are in urgent demand.^5^

Micro RNAs (miRNAs) are small, noncoding RNA molecules of ~19–25 nucleotides that potentially regulate 20–30 % of gene expression.^6^ Each miRNA has numerous targets, and depending on its target genes, a miRNA can play significant roles in tumor metastasis.^7^ In recent years, over 100 miRNAs have been implicated in CRC.^8^ However, more influential research is required to reveal how miRNAs act within the context of the molecular mechanisms for disease recurrence of CRC.

In this study, we conducted a microarray-based analysis to recognize differentially expressed miRNAs in CRC by comparing miRNA profiles among primary CRC tissues from patients without liver metastasis, primary tissues with liver metastasis, and liver metastatic lesions. After the miRNA array analysis, we evaluated the role of miR-132 in human CRC.

## Materials and Methods

### Collection of Human Tissue Specimens

The testing cohort consisted of 28 primary CRC lesions and eight metastatic liver tumors that arose from CRC. Sixteen primary CRC lesion samples were collected from patients with stage II and III disease without liver metastasis during the follow-up period (median 1898 days, range 27–2155 days) after operation. Twelve primary CRC lesion samples were from patients with concomitant liver metastasis. The extended independent validation cohort consisted of tissue samples from 151 patients [135 primary lesions (109 without liver metastasis and 26 with liver metastasis), and 16 liver metastatic lesions arising from CRC]. These 135 patients included patients with stage I (*n* = 30), stage II (*n* = 42), and stage III (*n* = 37) CRC. The median follow-up period for these 135 patients was 1653 days (range 27–2228 days). The clinical characteristics of the testing cohort and the validation cohort are summarized in Supplementary Table S1. To compare the expression of miRNA in tumor versus normal tissues, the primary CRC tissues (*n* = 21) and their adjacent normal tissues (*n* = 21) were assessed using quantitative real-time PCR (qRT-PCR). All patients were resected with curative intent between 2003 and 2013 at Osaka University Hospital and its three related hospitals. The samples were stored at −80 °C as a fresh frozen samples with RNAlater (Ambion, Austin, TX, USA) until RNA extraction. All patients provided written informed consent, in accordance with the guidelines approved by the institutional research board of each institute.

### RNA Extraction

Total RNA from tissues and cells were isolated using the miRNeasy Mini Kit (Qiagen, Hilden, Germany) and Trizol reagent (Invitrogen, Carlsbad, CA, USA) following the manufacturer’s protocol.

### Microarray Analysis

Microarray analysis (Sureprint G3 Humans miRNA 8 × 60 K; Agilent Technologies, Santa Clara, CA, USA) was performed for the testing cohort (*n* = 36) at Hokkaido System Science Corporation (Hokkaido, Japan) using miRNA Complete Labeling Reagent and Hyb Kit (Agilent Technologies). The microarray raw data are available in Gene Expression Omnibus (GEO; https://www.ncbi.nlm.nih.gov/geo/) database with accession code GSE72199.

### Reverse Transcription PCR and TaqMan miRNA Assay

In the TaqMan microRNA Assay (Applied Biosystems, Foster City, CA, USA), hsa-miR-132 ID 000457 and RNU6B ID 001093 were used to measure miRNA levels. The TaqMan MicroRNA Reverse Transcription kit and TaqMan 2× universal PCR Master Mix, No AmpErase UNG (Applied Biosystems), were used according to the manufacturer’s protocol. The 7900HT Sequence Detection System 2.3 (Applied Biosystems) software was used to compute the relative change in RNA expression by the $2^{{ - \varDelta \varDelta C_{\text{t}} }}$ method.

### qRT-PCR

Total RNA was reverse transcribed using the High Capacity RNA-to-cDNA Kit (Applied Biosystems). We performed qRT-PCR using the TaqMan Gene Expression Assay (Applied Biosystems) following the manufacturer’s protocol.

### Cell Lines and Cell Culture

All human CRC cell lines (DLD-1 and HCT116) were obtained from the American Type Culture Collection in 2001. Cell lines were cultured in Dulbecco’s modified Eagle medium (DMEM D6046; Sigma Aldrich, St. Louis, MO, USA) containing 10 % fetal bovine serum in a humidified incubator under 5 % CO_2_ at 37 °C.

### Cell Transfection

To perform transient transfections, the cells were transfected using Lipofectamine RNAiMAX (Invitrogen), with 25 nmol/L of miRVana miRNA mimic miR-132–3p (#4464066) and miRVana miRNA inhibitor miR-132–3p (#4464064), according to the manufacturer’s instructions. The control miRNAs [Negative Control #1 mimic (#4464058) and Negative Control #1 (#4464074)] were used as a control for nonspecific effects.

### Proliferation Assays

Cells were seeded at a density of 2.5–3 × 10^4^ per well in 24-well dishes and cultured for 72 h to determine proliferation. Cells were counted with a Celltac automatic hematology analyzer (Nihon Kohden, Tokyo, Japan).

### Colony Formation

For colony formation assays, 500 transfected cells were plated in six-well plates. After incubation at 37 °C for 11 days, visible colonies were fixed with formalin and stained with Giemsa solution, and the numbers of colonies were counted using a microscope in 10 random visual fields (×4 magnification, *n* = 4).

### Invasion Assay

The cell invasion assay was performed using transwell inserts with 8 μm pores (BD Biosciences, San Jose, CA, USA), in accordance with the manufacturer’s protocol. The invading cells were counted using a microscope in three random visual fields (×200 magnification).

### Luciferase Reporter Assay

We amplified the ANO1 3′ untranslated region (3′ UTR) containing the putative miR-132 binding sites by PCR using following primers: ANO1 (forward) 5′-GCTCGCTAGCCTCGAGGGGGCGTGGGAGCATCC-3′, (reverse) 5′-ATGCCTGCAGGTCGATGGCAGATTAAAGGGAATGT-3′. The resulting DNA fragment was inserted at restriction sites immediately downstream of the luciferase gene in the pmirGLO vector (Promega, Madison, WI, USA). Luciferase activity was measured using the Dual Luciferase Reporter Assay System (Promega). Firefly luciferase activity was normalized against *Renilla* luciferase activity for each transfected well.

### Statistical Analysis

Data were expressed as means ± standard deviations. Statistical analysis was performed using JMP Pro 10 software (SAS Institute, Cary, NC, USA). Statistical significance was compared using the Chi square test, and continuous variables were compared using Student’s *t* test or one-way analysis of variance, as appropriate. Survival curves were generated using the Kaplan–Meier method and assessed using the log-rank test. The Cox proportional hazard regression model was performed to identify independent prognostic factors. A value of *P* < 0.05 was considered statistically significant.

## Results

### Expression of miR-132 in Colorectal and Hepatic Tissue Samples

**Fig. 1:**
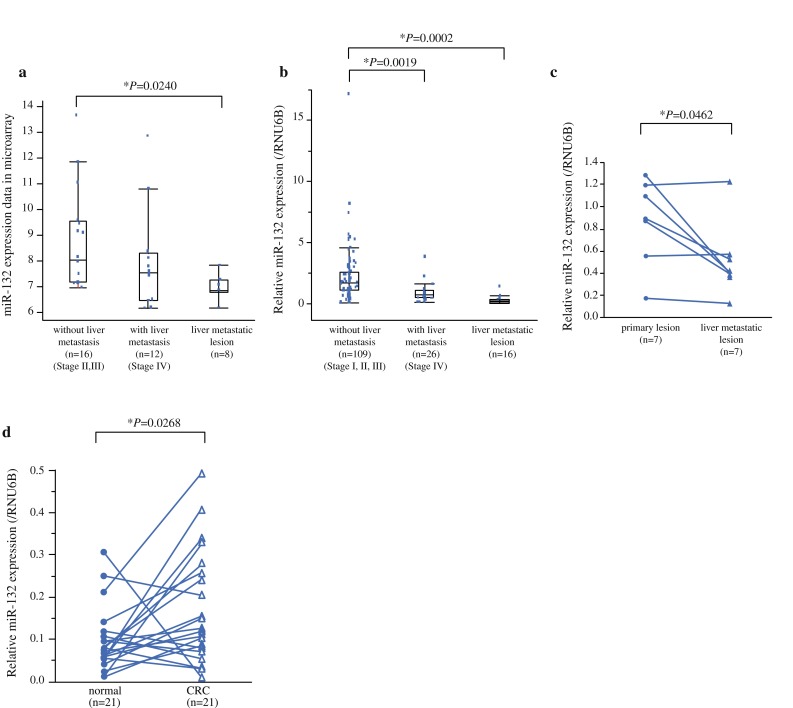
Expression of miR-132 in colorectal and hepatic tissue samples. **a** Microarray data showed that miR-132 expression was significantly lower in liver metastatic lesions than in primary CRC tumors from patients without liver metastasis. **b** In extended validation cohort, qRT-PCR showed that miR-132 expression was significantly down-regulated in primary CRC lesions with liver metastasis and in liver metastatic lesions compared to primary CRC lesions without liver metastasis. **c** MiR-132 expression of seven pairs of primary CRC was significantly down-regulated in corresponding synchronous liver metastases. **d** MiR-132 expression was significantly increased in tumor tissues (*n* = 21) compared to their pair-matched adjacent normal colon tissues (*P* = 0.0268)

To explore the possible role of miRNA in CRC, miRNA expression was profiled in the testing cohort, which consisted of primary CRC tumor tissue from patients without liver metastasis (*n* = 16), tumor tissue from patients with liver metastasis (*n* = 12), and liver metastatic lesions (*n* = 8). MiRNA array analysis identified 39 miRNAs (Supplementary Table S2) whose expression levels differed between primary CRC lesions without and with liver metastasis (>2-fold change). Among them, we found that a tumor suppressor miR-132 expression was significantly lower in CRC liver metastatic lesions than in primary CRC lesions without liver metastasis (Fig. 1a).^9^ The microarray results of miR-132 expression were validated by qRT-PCR in the same testing cohort of 36 CRC tissues. The expression of miR-132 was significantly higher in primary CRC lesions without and with liver metastasis than in liver metastatic lesions (Supplementary Fig. S1a). A statistically significant correlation was observed between microarray data and qRT-PCR miR-132 expression data (*r* = 0.549, *P* = 0.0005; Supplementary Fig. S1b).

On the basis of the preliminary data, we then examined miR-132 expression by qRT-PCR in the independent and extended validation cohort of 151 patients (109 primary CRC lesions without liver metastasis, 26 primary lesions with liver metastasis, and 16 liver metastatic lesions). As results, miR-132 expression was significantly down-regulated in primary CRC lesions with liver metastasis and in liver metastatic lesions compared to primary CRC lesions without liver metastasis (Fig. 1b, *P* = 0.0019 and *P* = 0.0002, respectively). In this study, we analyzed seven pairs of primary CRC and corresponding synchronous liver metastases that were collected from both microarray and validation cohort. MiR-132 expression was significantly down-regulated in corresponding liver metastases compared to primary CRC lesions (Fig. 1c, *P* = 0.0462). When we measured the difference expression between tumor and normal tissues, miR-132 expression was significantly increased in tumor tissues (*n* = 21) compared to their pair-matched adjacent normal colonic tissues (Fig. 1d, *P* = 0.0268).

### Overexpression of miR-132 Suppresses CRC Cell Growth and Invasion In Vitro

**Fig. 2:**
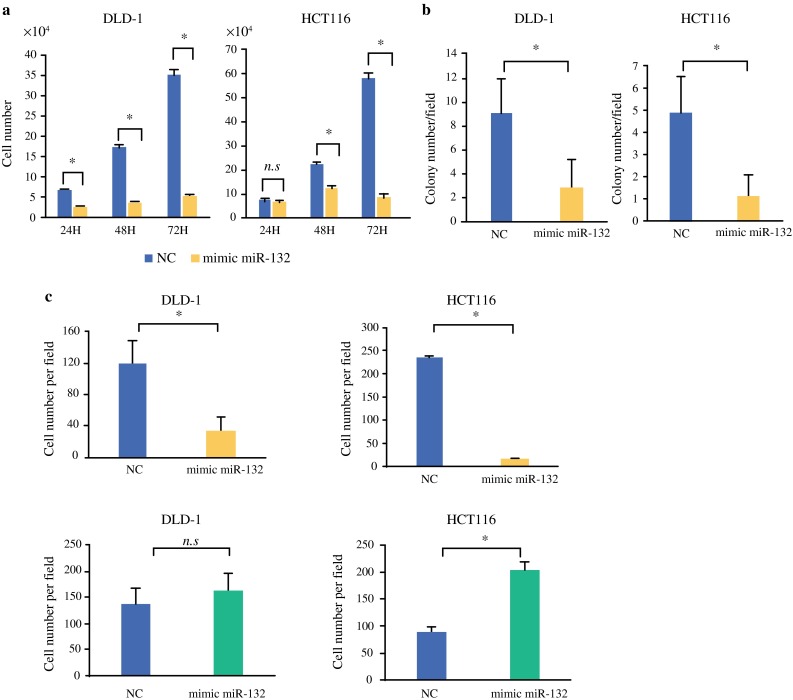
In vitro transduction of miR-132 in CRC cells. **a** MiR-132-transfected cells demonstrated significantly slower growth rate than negative control-transfected cells. **b** Colony formation assay showed that miR-132 overexpression resulted in significant inhibition of tumor growth. **c** MiR-132 overexpression markedly reduced invasion ability compared to negative control. In contrast, miR-132 inhibitor increased invasion ability. Data are presented as mean ± SD. *NC* negative control-transfected cells, *mimic miR*-*132* miR-132-transfected cells, *inhibitor miR*-*132* inhibitor miR-132-transfected cells. *P < 0.05

CRC cells were transfected with miR-132 mimics, and the transfection efficiency was measured through real-time PCR. There was significantly higher miR-132 expression in miR-132-transfected cells at every time point compared to negative control (NC) miR-transfected cells (Supplementary Fig. S2). MiR-132-transfected CRC cells demonstrated a significantly slower growth rate than NC (Fig. 2a). Results of the colony-formation assay indicated that miR-132 overexpression significantly inhibited tumor growth (Fig. 2b). Because cell invasion is an initial step of metastasis, we examined the effect of miR-132 on the invasive capacity of CRC cell lines. MiR-132 overexpression markedly reduced the invasion ability compared to NC (Fig. 2c). In contrast, inhibiting miR-132 expression (inhibitor miR-132) showed increased invasion ability compared to NC (Fig. 2c).

### MiR-132 Directly Targets 3′ UTR of ANO1 in CRC Cells

**Fig. 3:**
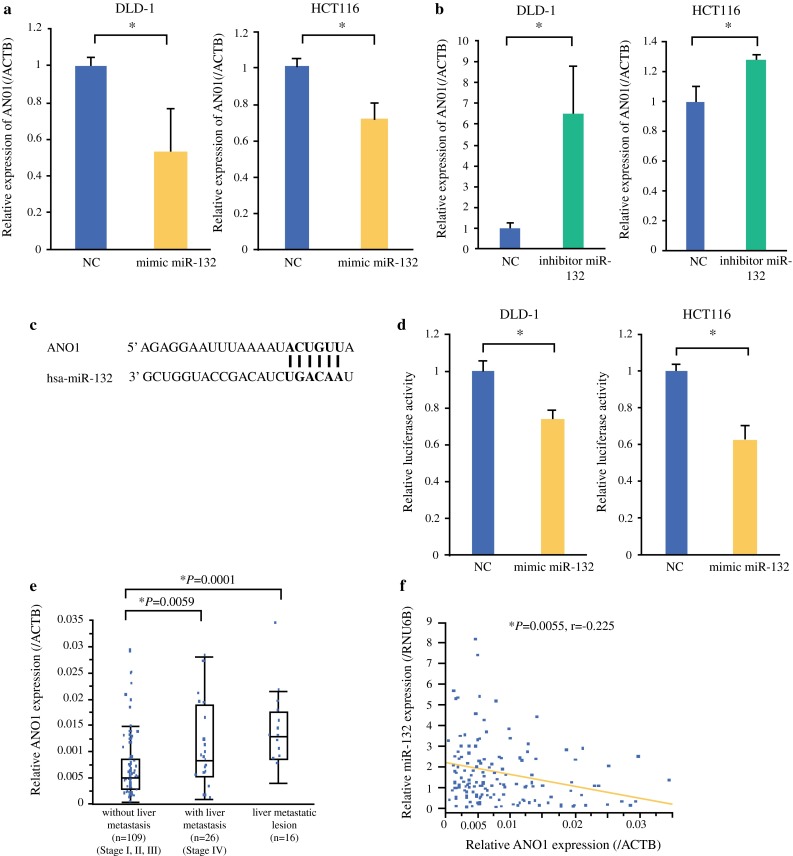
ANO1 as possible target of miR-132. **a** MiR-132 expression markedly suppressed ANO1 mRNA levels. **b** MiR-132 inhibitor resulted in up-regulation of ANO1 mRNA levels. **c** The 3′ UTR of ANO1 mRNA contains site for seed region of miR-132. **d** Activity of luciferase reporter containing predicted miR-132 binding sequence of ANO1 3′ UTR was significantly repressed by ectopic expression of miR-132. **e** ANO1 expression was higher in primary CRC with liver metastasis and in liver metastases than in primary tumors without metastasis (*P* = 0.0059 and *P* = 0.0001, respectively). **f** Significant inverse correlation was observed between miR-132 expression and ANO1 expression in validation cohort. Data are presented as mean ± SD. *NC* negative control-transfected cells, *mimic miR*-*132* miR-132-transfected cells, *inhibitor miR*-*132* inhibitor miR-132-transfected cells. *P < 0.05

To reveal the biological potential role of miR-132 in CRC, we explored the targets of miR-132. Using 390 expression arrays of CRC in the GEO database (GSE41258) and the target prediction tool TargetScan 6.2, we predicted the potential target genes. We identified 54 genes (Supplementary Table S3) and focused ANO1 as a potential target of miR-132. MiR-132 overexpression markedly suppressed ANO1 mRNA expression levels (Fig. 3a), while miR-132 inhibitor resulted in the up-regulation of ANO1 mRNA expression levels (Fig. 3b). The 3′ UTR of ANO1 mRNA contains a complementary site for the seed region of miR-132 (Fig. 3c). More specifically, in order to show that ANO1 is a direct target of miR-132, we cotransfected miR-132 expression vector along with the ANO1 3′ UTR sequence containing luciferase reporter constructs. The activity of a luciferase reporter containing the predicted miR-132 binding sequence of ANO1 3′ UTR was significantly repressed by the ectopic expression of miR-132 (Fig. 3d).

### Up-regulation of ANO1 Is Inversely Associated with Down-regulation of miR-132 in CRC Clinical Samples

The above results prompted us to investigate whether miR-132 suppresses CRC growth and metastasis through ANO1 suppression. To further confirm the clinical relationship between miR-132 and ANO1 in CRC patients, we utilized qRT-PCR to examine ANO1 expression in the same validation cohort of 151 tissue samples that were used to examine miR-132 expression. ANO1 expression was higher in primary CRC with liver metastasis and in liver metastases than in primary tumors without metastasis (*P* = 0.0059, and *P* = 0.0001, respectively) (Fig. 3e). There was no significant difference in expression between tumors with liver metastasis and liver metastasis. Moreover, we found a significant inverse correlation between miR-132 expression levels and ANO1 expression levels in the validation cohort (*r* = −0.225 *P* = 0.0055; Fig. 3f).

### MiR-132 Acts as Prognostic Marker of CRC

**Fig. 4:**
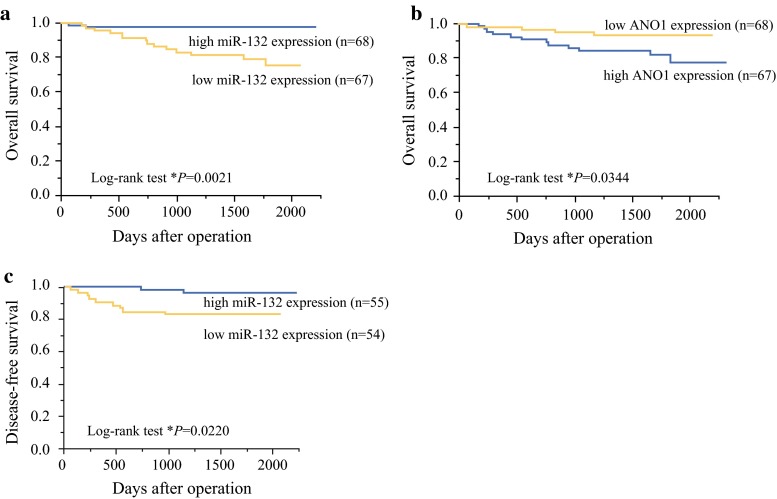
MiR-132 acts as prognostic marker for CRC. **a** Kaplan–Meier survival curves showed that patients with low miR-132 expression demonstrated poorer clinical outcome (OS, *P* = 0.0021, median follow-up 1653 days). **b** Patients with low ANO1 levels had more favorable clinical outcome (OS) than patients with high ANO1 levels. **c** DFS rate were significantly lower in patients with low miR-132 expression than in patients with high miR-132 expression (*P* = 0.0220)

The clinicopathologic implications of miR-132 expression were assessed in 135 CRC patients from the validation cohort of primary CRC tissue samples. To clarify the correlation of miR-132 expression and postoperative survival of patients, we divided the patients into two groups according to the median value (1.299) of the miR-132 expression. Low miR-132 expression was positively associated with tumor size, depth, lymph node metastasis, venous permeation, and clinical disease stage (Supplementary Table S4). Kaplan–Meier survival curves showed that patients with low miR-132 expression demonstrated significantly worse clinical outcome [overall survival (OS): log-rank test *P* = 0.0021; median follow-up: 1653 days; Fig. 4a]. Univariate analysis for OS revealed that tumor depth, lymph node metastasis, lymphatic permeation, venous permeation, clinical stage, and miR-132 expression were significantly associated with OS (Table 1). Multivariate analysis for OS indicated that miR-132 expression (relative risk 3.838, 95 % confidence interval 1.054–24.683, *P* = 0.040), lymphatic permeation, and clinical stage were independent prognostic factors for CRC patients (Table 2).

**Table 1:** Univariate and multivariate analyses for overall survival

Characteristic	Univariate analysis		*P*	Multivariate analysis		*P*
	RR	95 % CI		RR	95 % CI	
Sex						
Male/female	0.586	0.215–1.593	0.288			
Lesion						
Colon/rectum	0.783	0.288–2.129	0.626			
Differentiation						
tub1, tub2/muc, por	0.302	0.084–1.933	0.173			
Tumor size						
≤35 mm/>35 mm	0.566	0.130–1.756	0.347			
Depth						
T1, T2/T3, T4	<0.0001	0.386–0.386	0.003*	<0.0001	<0.0001–2.574	0.192
Lymph node metastasis						
Negative/positive	0.156	0.036–0.483	0.001*	1.852	0.409–6.243	0.383
Lymphatic permeation						
Negative/positive	0.064	0.004–0.315	<0.0001*	0.103	0.006–0.537	0.004*
Venous permeation						
Negative/positive	0.277	0.064–0.861	0.025*	0.890	0.196–2.999	0.860
Stage						
I, II/III, IV	<0.0001	0.0968–0.0968	<0.0001*	<0.0001	<0.0001–0.218	0.002*
miR-132 expression						
Low/high	7.255	2.026–46.207	0.001*	3.838	1.054–24.683	0.040*

*RR* relative risk, *CI* confidence interval

* Statistically significant

**Table 2:** Univariate and multivariate analyses for disease-free survival

Characteristic	Univariate analysis			Multivariate analysis		
	RR	95 % CI	*P*	RR	95 % CI	*P*
Sex						
Male/female	0.978	0.295–3.733	0.972			
Lesion						
Colon/rectum	0.697	0.201–2.314	0.550			
Differentiation						
tub1, tub2/muc, por	0.148	0.038–0.971	0.047*	0.099	0.016–0.765	0.030*
Tumor size						
≤35 mm/>35 mm	0.235	0.013–1.227	0.094			
Depth						
T1, T2/T3, T4	<0.0001	0.429–0.429	0.005*	<0.0001	0.269–1.201	0.071
Lymph node metastasis						
Negative/positive	0.179	0.039–0.620	0.006*	0.331	0.071–1.183	0.090
Lymphatic permeation						
Negative/positive	0.286	0.063–0.990	0.048*	0.277	0.060–0.978	0.046*
Venous permeation						
Negative/positive	0.767	0.221–2.547	0.661			
miR-132 expression						
Low/high	5.01	1.290–32.834	0.018*	5.838	1.374–42.770	0.015*

*RR* relative risk, *CI* confidence interval

* Statistically significant

To examine the association of ANO1 expression with clinical outcome, we divided the patients into two groups according to the median value (0.0055) of the ANO1 expression. Kaplan–Meier survival curves revealed that patients with low ANO1 expression had a more favorable clinical outcome (OS) than patients with high ANO1 expression (log-rank test *P* = 0.0344, Fig. 4b). These results suggest that ANO1 up-regulation through miR-132 suppression might affect the clinical outcome (OS) of patients with CRC.

In 109 samples from patients with CRC (excluding 26 stage IV CRCs with liver metastasis), disease-free survival (DFS) rate were significantly lower in patients with low miR-132 expression than in patients with high miR-132 expression (log-rank test *P* = 0.0220, Fig. 4c). Low miR-132 expression was positively associated with large tumor size and deep invasion (Supplementary Table S5). In addition, univariate and multivariate analysis showed that miR-132 expression was an independent risk factor for DFS (*P* = 0.015; median follow-up: 1659 days; Table 2) in CRC patients. On the other hand, ANO1 expression was not a significant parameter for DFS (data not shown).

## Discussion

Recent miRNA studies have highlighted cancer invasion and metastasis. Following microarray study, miR-214 and miR-181a were already suggested as regulators of CRC liver metastasis.^10^,^11^ In the present study, we examined miRNA profiles in primary CRC lesions with and without liver metastasis and in liver metastatic lesions to identify the key miRNA related to CRC.

On the basis of the microarray data, we validated the miR-132 expression in a larger independent validation cohort. MiR-132 was definitely down-regulated in primary CRC lesions with liver metastasis and also in liver metastatic lesions. Furthermore, down-regulation of miR-132 was associated with poorer OS and DFS in patients with CRC.

MiR-132 has been reported as a tumor suppressor in a series of cancers.^9^,^12^–^15^ Zheng et al. showed that miR-132 inhibits CRC invasion and metastasis via directly targeting ZEB2 using 62 CRC samples.^15^ Our study extended their findings with a possible target ANO1 using a large scale of CRC samples (*n* = 163) as well as their metastatic lesions to liver (*n* = 24). Of interest was that low miR-132 expression was still maintained in hepatic metastatic lesions (Fig. 1b). Moreover, direct comparison of seven paired samples between primary CRC tumor and its corresponding synchronous hepatic metastases showed that hepatic metastases had even smaller amount of miR-132 compared to primary CRC tumors (Fig. 1c), although the paired number was limited in this study. From a therapeutic point of view, therefore, it is worth exploring whether mimic miR-132 would inhibit liver metastasis in animal models. Further study would clarify more about the close link of miR-132 with liver metastasis.

When we compared miR-132 levels between normal and tumor tissues, we found that miR-132 expression was even higher in tumor tissues than in normal tissues. This result appears to be paradoxical to our finding of down-regulation of miR-132 in advanced or metastatic stage tumors. In this regard, Kara et al. showed a supportive result that miR-132 in CRC tissues had approximately threefold increase of that in normal mucosa.^16^ Therefore, we postulate that miR-132 might have differential roles in carcinogenesis and in tumor progression, although further studies are required to reach a definitive conclusion.

Each miRNA can potentially down-regulate many target genes by binding their 3′ UTRs. Our results showed that ANO1 is a target of miR-132 that has a crucial role in CRC progression. ANO1 is also known as discovered on gastrointestinal stromal tumor protein (DOG1) and tumor-amplified and overexpressed sequence 2 (TMEM16A), and it is highly deregulated in different human cancers.^17^–^19^ These studies showed that ANO1 was a critical oncogenic factor that contributed to cell motility, invasion, and adhesion. Furthermore, the overexpression of ANO1 has a significant effect on both distant metastasis and poor prognosis. Our results suggest a potential role for ANO1 in CRC through miR-132. ANO1, which consists of 26 exons and encodes a protein that contains eight transmembrane regions, acts as a calcium-activated chloride channel.^20^–^22^ Calcium-activated chloride channels have recently become notable as a new drug target for anticancer therapy.^23^ ANO1 may contribute to this potential new approach to cancer therapy.

In conclusion, we demonstrated that lower expression of miR-132 in clinical CRC specimens was associated with CRC progression and poor survival. The tumor-suppressing function of miR-132 may in part be realized through targeting of the downstream gene ANO1. Our data suggest that further study is essentially important to reveal the relation between miR-132 and liver metastasis as well as the potential of miR-132 as a therapeutic target of CRC.

## Electronic supplementary material

Below is the link to the electronic supplementary material.

Supplementary Fig. S1 Validation of microarray data of the preliminary cohort by qRT-PCR. (a) qRT-PCR showed that the relative expression of miR-132 was significantly higher in primary CRC lesions without and with liver metastasis than in liver metastatic lesions. (b) There is a significant correlation between microarray data and qRT-PCR expression data. Supplementary material 1 (TIFF 1429 kb)

Supplementary Fig. S2 Transfection efficiency data. The data are presented as the mean ± SD. (NC; negative control-transfected cells, mimic miR-132; miR-132-transfected cells, *P<0.05). Supplementary material 2 (TIFF 7302 kb)

Supplementary material 3 (DOCX 27 kb)
