# Supplementary figures and images for: Down-Regulation of microRNA-132 is Associated with Poor Prognosis of Colorectal Cancer
Source: Ann Surg Oncol. 2016 Feb 11;23(Suppl 5):599–608. doi: 10.1245/s10434-016-5133-3 (PMC5149564; doi:10.1245/s10434-016-5133-3)

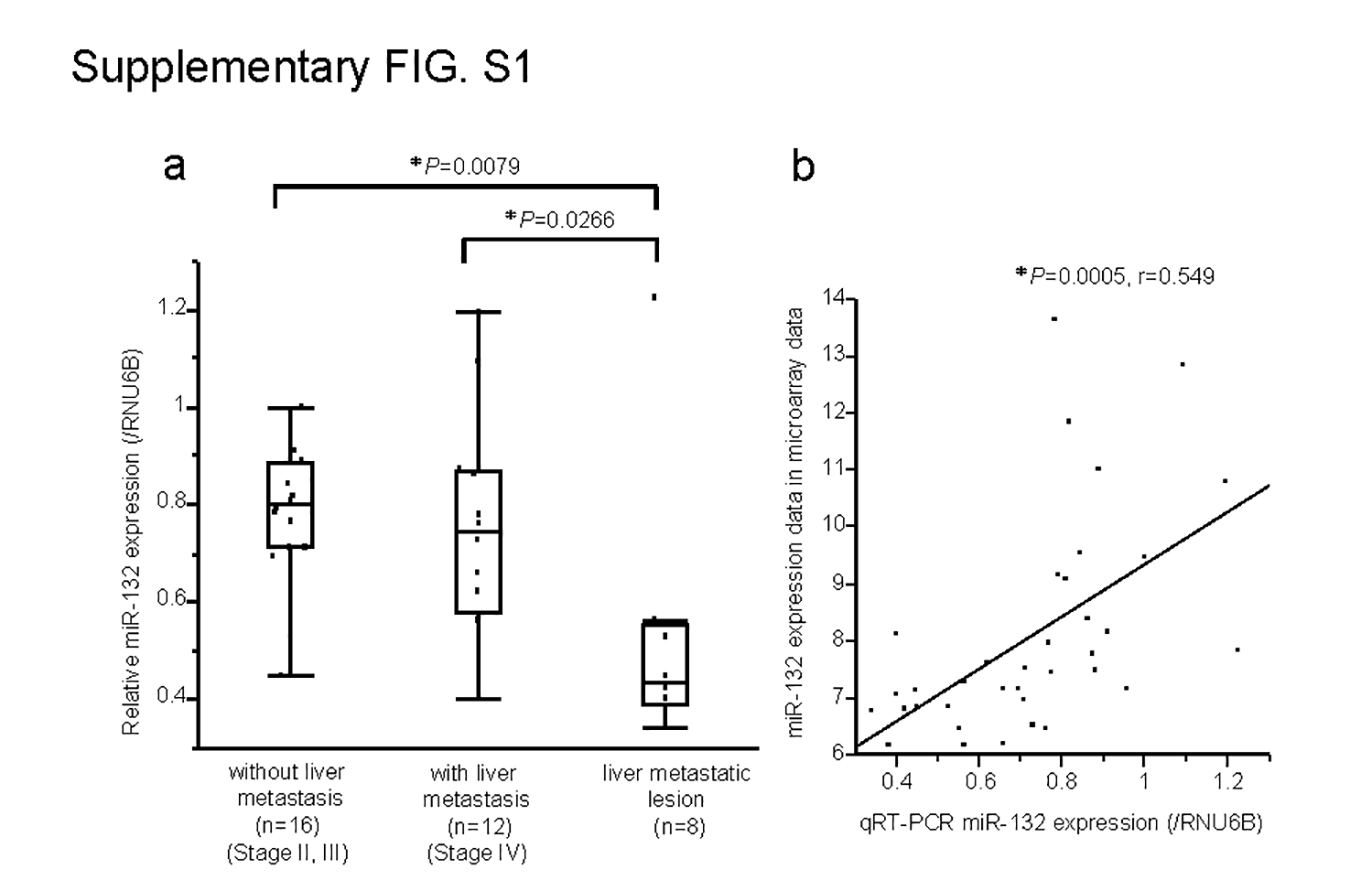

Supplement: Supplementary file 1 — Validation of microarray data of the preliminary cohort by qRT-PCR. (a) qRT-PCR showed that the relative expression of miR-132 was significantly higher in primary CRC lesions without and with liver metastasis than in liver metastatic lesions. (b) There is a significant correlation between microarray data and qRT-PCR expression data. Supplementary material 1 (TIFF 1429 kb) [file 10434_2016_5133_MOESM1_ESM.tif]

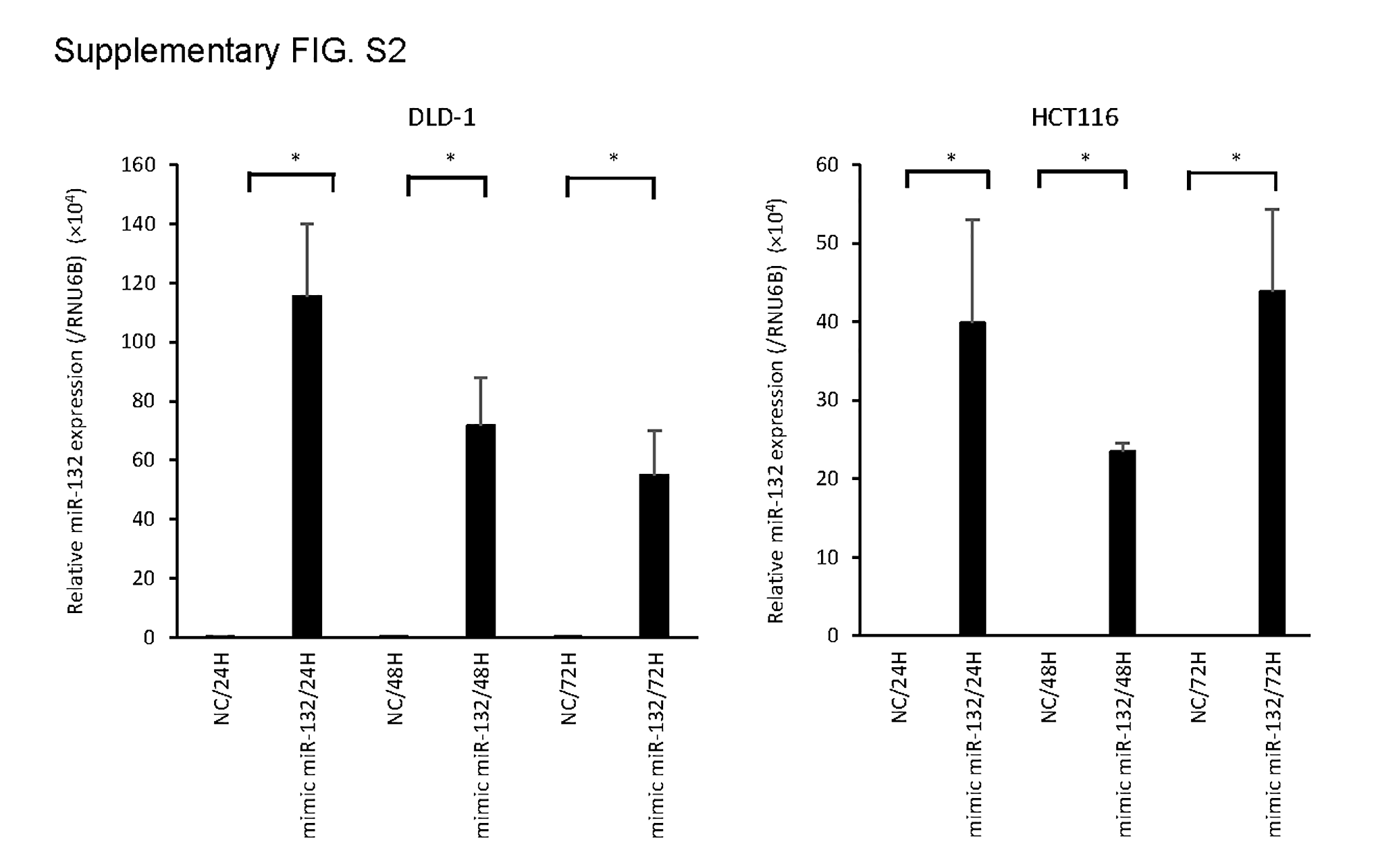

Supplement: Supplementary file 2 — Transfection efficiency data. The data are presented as the mean ± SD. (NC; negative control-transfected cells, mimic miR-132; miR-132-transfected cells, *P<0.05). Supplementary material 2 (TIFF 7302 kb) [file 10434_2016_5133_MOESM2_ESM.tif]
