# Supplementary material for: Down-Regulation of microRNA-132 is Associated with Poor Prognosis of Colorectal Cancer
Source: Ann Surg Oncol. 2016 Feb 11;23(Suppl 5):599–608. doi: 10.1245/s10434-016-5133-3 (PMC5149564; doi:10.1245/s10434-016-5133-3)
Supplement: Supplementary file 3 — Supplementary material 3 (DOCX 27 kb) [file 10434_2016_5133_MOESM3_ESM.docx]

Supplementary Table S1

Characteristics of the testing cohort and the validation cohort

|  | Testing cohort | | Validation cohort | |
| --- | --- | --- | --- | --- |
| Patient characteristics | Without liver metastasis  (Stage II/III) | With liver metastasis | Without liver metastasis  (Stage I/II/III) | With liver metastasis |
| Number of patients | 16 | 12 | 109 (30/42/37) | 26 |
| Sex |  |  |  |  |
| Male | 8 | 5 | 70 | 15 |
| Female | 8 | 7 | 39 | 11 |
| Lesion |  |  |  |  |
| Colon | 7 | 6 | 60 | 15 |
| Rectum | 9 | 6 | 49 | 11 |
| Differentiation |  |  |  |  |
| tub1, tub2 | 15 | 11 | 104 | 24 |
| muc, por | 1 | 1 | 5 | 2 |
| Tumor size |  |  |  |  |
| ≤35 mm | 6 | 3 | 32 | 6 |
| >35 mm | 10 | 9 | 77 | 20 |
| Depth |  |  |  |  |
| T1, T2 | 1 | 0 | 33 | 0 |
| T3, T4 | 15 | 12 | 76 | 26 |
| Lymph node metastasis |  |  |  |  |
| Negative | 8 | 5 | 72 | 5 |
| Positive | 8 | 7 | 37 | 21 |
| Lymphatic permeation |  |  |  |  |
| Negative | 1 | 2 | 61 | 4 |
| Positive | 15 | 10 | 48 | 22 |
| Venous permeation |  |  |  |  |
| Negative | 6 | 0 | 56 | 4 |
| Positive | 10 | 12 | 53 | 22 |

Supplementary Table S2

Microarray analysis

| hsa-miR-1227 |
| --- |
| hsa-miR-1288 |
| hsa-miR-132 |
| hsa-miR-139-3p |
| hsa-miR-142-3p |
| hsa-miR-142-5p |
| hsa-miR-150 |
| hsa-miR-204 |
| hsa-miR-212 |
| hsa-miR-3125 |
| hsa-miR-3127 |
| hsa-miR-3132 |
| hsa-miR-3138 |
| hsa-miR-3149 |
| hsa-miR-3154 |
| hsa-miR-3156 |
| hsa-miR-3188 |
| hsa-miR-3202 |
| hsa-miR-340 |
| hsa-miR-3607-3p |
| hsa-miR-374c |
| hsa-miR-4312 |
| hsa-miR-4317 |
| hsa-miR-454 |
| hsa-miR-487b |
| hsa-miR-490-5p |
| hsa-miR-494 |
| hsa-miR-498 |
| hsa-miR-500a |
| hsa-miR-557 |
| hsa-miR-563 |
| hsa-miR-582-3p |
| hsa-miR-605 |
| hsa-miR-623 |
| hsa-miR-625 |
| hsa-miR-652 |
| hsa-miR-664 |
| hsa-miR-887 |
| hsa-miR-92b |

Supplementary Table S3

Potential target genes of miR-132

| CDK19 | FXR1 |
| --- | --- |
| TMEM106B | NMT2 |
| LRRFIP1 | EIF4A2 |
| SOX5 | IQSEC1 |
| SLC6A1 | RAB6B |
| GAPVD1 | HAO1 |
| DYNC1LI2 | ARHGEF40 |
| ACVR2B | PHF20L1 |
| FOXO3 | ZNF292 |
| AMOT | SOX4 |
| SETD5 | CDC42BPA |
| ARHGEF11 | FOXN3 |
| MTMR10 | RAPGEF5 |
| DCC | ARHGAP5 |
| INTS6 | ANO1 |
| VPS41 | NFAT5 |
| ProSAPiP1 | PDE7B |
| HIC2 | CCNT2 |
| CELSR3 | JHDM1D |
| ASB1 | UBN1 |
| ARFGAP2 | CUX1 |
| ZNF362 | MECP2 |
| SLC2A1 | SLC1A2 |
| STX16 | GSK3B |
| TCF7L1 | SHANK2 |
| KLHDC10 | KIF21B |
| DCAF8 | PHIP |

Supplementary Table S4

Correlation between miR-132 expression and clinicopathological characteristics in colorectal cancer patients (overall survival)

| Patient characteristics | miR-132 expression | | *P* |
| --- | --- | --- | --- |
|  | High (n=68) | Low (n=67) |  |
| Sex |  |  |  |
| Male | 45 | 40 |  |
| Female | 23 | 27 | 0.436 |
| Lesion |  |  |  |
| Colon | 37 | 38 |  |
| Rectum | 31 | 29 | 0.788 |
| Differentiation |  |  |  |
| tub1, tub2 | 65 | 63 |  |
| muc, por | 3 | 4 | 0.683 |
| Tumor size |  |  |  |
| ≤35 mm | 25 | 13 |  |
| >35 mm | 43 | 54 | 0.025* |
| Depth |  |  |  |
| T1, T2 | 25 | 8 |  |
| T3, T4 | 43 | 59 | 0.001* |
| Lymph node metastasis |  |  |  |
| Negative | 48 | 29 |  |
| Positive | 20 | 38 | 0.001* |
| Lymphatic permeation |  |  |  |
| Negative | 32 | 33 |  |
| Positive | 36 | 34 | 0.799 |
| Venous permeation |  |  |  |
| Negative | 38 | 22 |  |
| Positive | 30 | 45 | 0.007* |
| Stage |  |  |  |
| I, II | 46 | 26 |  |
| III, IV | 22 | 41 | 0.001* |

*** statistically significant

Supplementary Table S5

Correlation between miR-132 expression and clinicopathological characteristics in colorectal cancer patients (disease-free survival)

| Patient characteristics | miR-132 expression | | *P* |
| --- | --- | --- | --- |
|  | High (n=55) | Low (n=54) |  |
| Sex |  |  |  |
| Male | 36 | 34 |  |
| Female | 19 | 20 | 0.786 |
| Lesion |  |  |  |
| Colon | 28 | 32 |  |
| Rectum | 27 | 22 | 0.381 |
| Differentiation |  |  |  |
| tub1, tub2 | 52 | 52 |  |
| muc, por | 3 | 2 | 0.662 |
| Tumor size |  |  |  |
| ≤35 mm | 24 | 8 |  |
| >35 mm | 31 | 46 | 0.001* |
| Depth |  |  |  |
| T1, T2 | 22 | 11 |  |
| T3, T4 | 33 | 43 | 0.026* |
| Lymph node metastasis |  |  |  |
| Negative | 41 | 31 |  |
| Positive | 14 | 23 | 0.059 |
| Lymphatic permeation |  |  |  |
| Negative | 26 | 35 |  |
| Positive | 29 | 19 | 0.065 |
| Venous permeation |  |  |  |
| Negative | 33 | 23 |  |
| Positive | 22 | 31 | 0.069 |
| Stage |  |  |  |
| I, II | 41 | 31 |  |
| III | 14 | 23 | 0.059 |

*** statistically significant
